# Transcriptome analysis of *MBD5*-associated neurodevelopmental disorder (MAND) neural progenitor cells reveals dysregulation of autism-associated genes

**DOI:** 10.1038/s41598-021-90798-z

**Published:** 2021-05-28

**Authors:** Sureni V. Mullegama, Steven D. Klein, Stephen R. Williams, Jeffrey W. Innis, Frank J. Probst, Chad Haldeman-Englert, Julian A. Martinez-Agosto, Ying Yang, Yuchen Tian, Sarah H. Elsea, Toshihiko Ezashi

**Affiliations:** 1 Department of Molecular and Human Genetics, Baylor College of Medicine, One Baylor Plaza, Houston, TX 77030 USA; 2 Department of Human Genetics, David Geffen School of Medicine, University of California, Los Angeles, Los Angeles, CA USA; 3 10X Genomics, San Francisco, CA USA; 4 Departments of Human Genetics, Pediatrics and Internal Medicine, University of Michigan, Ann Arbor, MI 48109 USA; 5 Mission Fullerton Genetics Center, Asheville, NC 28803 USA; 6 Department of Molecular Pharmacology and Physiology, University of South Florida, Tampa, FL 33612 USA; 7 Division of Animal Sciences and Bond Life Sciences Center, University of Missouri, Columbia, MO 65211 USA; 8 Present Address: Department of Molecular and Cellular Biology, College of Osteopathic Medicine, Sam Houston State University, Conroe, TX 77304 USA; 9 Present Address: Department of Pediatrics, Children’s Hospital of Philadelphia, Philadelphia, PA 19104 USA; 10 Present Address: Department of Medical Genetics, Children’s Hospital of Philadelphia, Philadelphia, PA 19104 USA

**Keywords:** Genetics, Neuroscience, Stem cells, Neurology

## Abstract

*MBD5*-associated neurodevelopmental disorder (MAND) is an autism spectrum disorder (ASD) characterized by intellectual disability, motor delay, speech impairment and behavioral problems; however, the biological role of methyl-CpG-binding domain 5, MBD5, in neurodevelopment and ASD remains largely undefined. Hence, we created neural progenitor cells (NPC) derived from individuals with chromosome 2q23.1 deletion and conducted RNA-seq to identify differentially expressed genes (DEGs) and the biological processes and pathways altered in MAND. Primary skin fibroblasts from three unrelated individuals with MAND and four unrelated controls were converted into induced pluripotent stem cell (iPSC) lines, followed by directed differentiation of iPSC to NPC. Transcriptome analysis of MAND NPC revealed 468 DEGs (q < 0.05), including 20 ASD-associated genes. Comparison of DEGs in MAND with SFARI syndromic autism genes revealed a striking significant overlap in biological processes commonly altered in neurodevelopmental phenotypes, with TGFβ, Hippo signaling, DNA replication, and cell cycle among the top enriched pathways. Overall, these transcriptome deviations provide potential connections to the overlapping neurocognitive and neuropsychiatric phenotypes associated with key high-risk ASD genes, including chromatin modifiers and epigenetic modulators, that play significant roles in these disease states.

## Introduction

Autism spectrum disorder (ASD) is an encompassing term that describes neurodevelopmental disorders that have common deficits in communication and social interactions, accompanied by stereotyped behaviors and restricted interest. The exact molecular mechanism(s) that leads to this clinical entity is unknown. Studies suggest that between 20 and 45% of all ASDs are associated with rare pathogenic variants in specific genes^1,2^. The use of chromosomal microarray (CMA) is advocated as the first tier genetic test for individuals with ASD^3,4^.

The utilization of CMA led to the identification of *MBD5-*associated neurodevelopmental disorder (MAND, MIM 156200), which is an ASD characterized by intellectual disability, motor delay, severe speech impairment, and behavioral problems^5^. MAND serves as an umbrella term that encompasses 2q23.1 deletion syndrome, *MBD5* variant cases, and 2q23.1 duplications^5^. The conditions in this group of disorders have copy number variations or pathogenic sequence variants in the methyl-binding domain 5 gene (*MBD5,* MIM 611472), which is responsible for the majority of the phenotypes present in MAND^6,7^. *MBD5* variants have been associated with ASD, epilepsy, and schizophrenia in multiple cohorts^7,8^. The pathological mechanisms and pathways underlying the primarily neurological, neurodevelopmental, and neurobehavioral aspects of the MAND phenotype are not well delineated. Deficiency of *Mbd5* in mice resulted in reduced body weight motor deficits, abnormal social behavior, impaired learning, and iron metabolism abnormalities^9,10^. However, the importance of the genes associated with MAND in specific neurodevelopmental functions remains largely undefined.

Utilization of animal models to understand autism and other human neurodevelopmental or psychiatric disorders proves to be difficult^11^. Further, the absence of the “perfect” animal model leads to the inability to truly understand the human pathological mechanism which can lead to potential therapeutic targets^12,13^. Recent studies utilizing stem cell technology to derive neurons has served as the popular method to model neurodevelopmental disorders and has revealed deeper understanding of the phenotype of these disorders^13–15^. In addition, next-generation RNA-sequencing (RNA-seq) has been utilized in numerous studies and is a valid tool to identify therapeutic targets, biomarkers, pathways, and genes associated with ASD modeled by stem cell-derived human neurons^16–19^.

In this study, we explore the underpinnings of the neurodevelopmental phenotype of MAND by creating neural progenitor cells (NPC) derived from patients with 2q23.1 deletion syndrome, followed by RNA-seq to identify the contributing molecular pathways. Data show that haploinsufficiency of one or more genes the 2q23.1 region leads to *FOXG1* upregulation in NPC, accompanied with differentially expressed genes implicated in forebrain regionalization in early neurogenesis. Altered genes significantly overlap with ASD-associated genes, providing further insight into mechanisms underlying ASD by identifying shared network perturbances caused by the genetically penetrant MAND-associated 2q23.1 deletion.

## Materials and methods

This study was approved by the Baylor College of Medicine Institutional Review Board. All samples and information were collected after obtaining written informed consent. Whole blood and skin samples were provided by each participant. Participant recruitment, sample collection, and all experiments were performed in accordance with relevant guidelines and regulations by the Baylor College of Medicine Institutional Review Board and associated regulatory committees.

### Primary cell culture

**Figure 1:**
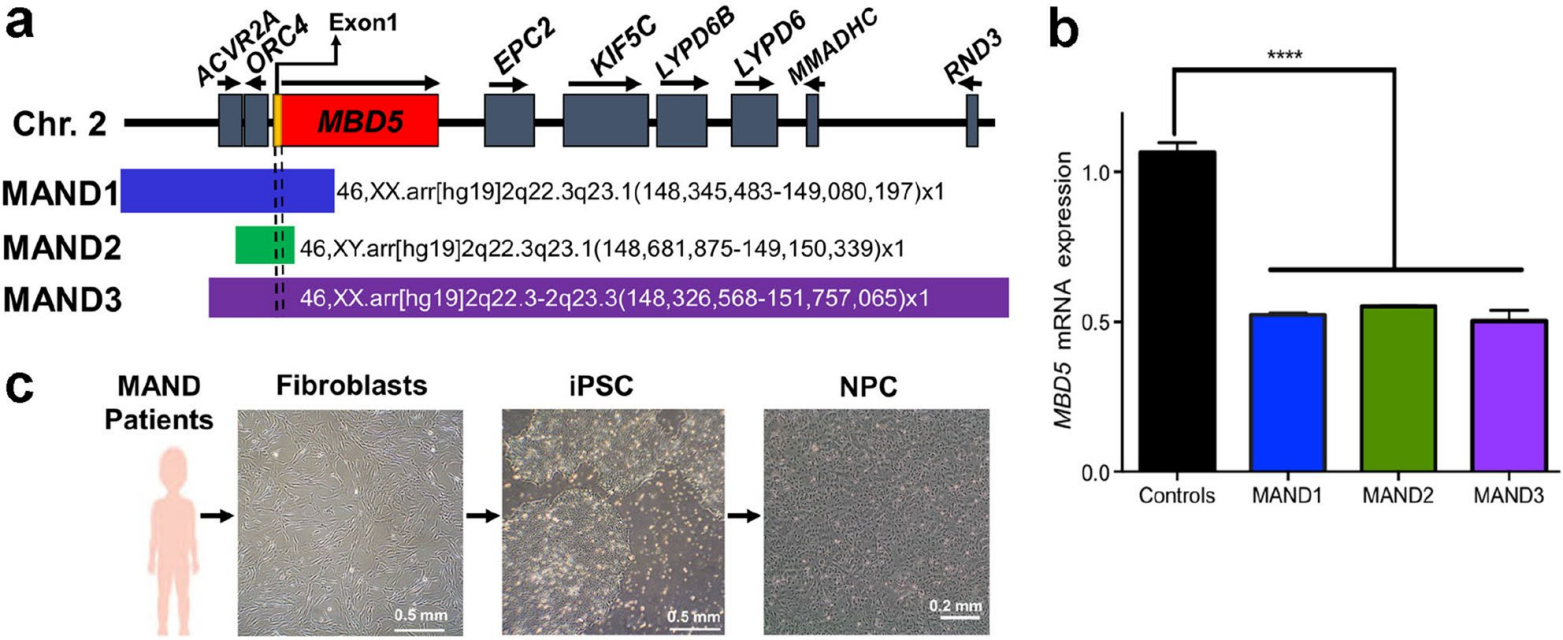
MAND patient 2q23.1 deletions include *MBD5* and exhibit reduced *MBD5* expression indicating haploinsufficiency. (**a**) Schematic diagram of the chromosome 2q23.1 deletion region. The coding region of *MBD5* is indicated by the red box; yellow region indicates non-coding exons. Chromosome 2q23.1 deletions present in the MAND patients in this study are summarized with bars. Additional details regarding MAND patients are provided in Supplementary Table 1. (**b**) qRT-PCR analysis showed ~ 50% reduction of *MBD5* mRNA expression in primary fibroblasts derived the three MAND patients compared to the controls. Expression of *MBD5* was calculated relative to that of the housekeeping gene, *GAPDH*. All expression values were calculated relative to control levels set at 1.0. Error bars represent the SEM. ****p < 0.0001, n = 3 independent biological replicates performed in triplicate, GraphPad Prism 8.4.2 (graphpad.com). (**c**) Overview of iPSC and neural progenitor cell (NPC) line derivations in this study. Skin fibroblasts from MAND patients (bar, 0.5 mm) were converted into iPSC by transfection with episomal plasmids encoding reprogramming factors (bar, 0.5 mm). NPC were derived from iPSC with neuronal induction medium (bar, 0.2 mm).

Primary skin fibroblast cells were derived and cultured in DMEM (Thermo) containing 10% FBS, 1% non-essential amino acids, 2 mM glutamine, 0.1 mM 2-mercaptoethanol, and 4 ng/ml human recombinant FGF2 (in house)^20^. Fibroblasts and NPC lines utilized in this study were obtained from four controls (CTR1, CTR2^21^, CTR3, CTR4) and three MAND patients with confirmed molecular diagnosis of 2q23.1 deletion syndrome by chromosomal microarray (Fig. 1a, Supplementary Table 1)^22–24^. MAND1 (SMS388, 3 y old female) has a 0.73 Mb loss at 2q22.3q23.1, 46,XX.arr[hg19]2q22.3q23.1(148,345,483–149,080,197) × 1,15q11.1q11.2(20,262,223–22,285,757) × 1^6,25^. MAND2 (SMS447, 10 y male) has a 0.18 Mb 2q23.1 deletion, 46,XY.arr[hg19]2q23.1(148,687,657–148,872,174) × 1. MAND3 (SMS456, 8 y female) has a 3.43 Mb deletion at 2q23.1, 46,XX.arr[hg19]2q22.3-q23.3(148,326,568–151,757,065) × 1^25^. All MAND cases were confirmed to have haploinsufficiency of *MBD5* (Fig. 1b).

### Reprogramming skin fibroblasts into iPSC with episomal plasmids

Early passages (between passages p3 and p5) of skin fibroblasts were cultured at low oxygen atmosphere of 4% O_2_/5% CO_2_/91% N_2_ and maintained in the condition for the entire reprograming period to enhance iPSC generation^21,26^. Episomal vectors carrying shRNA for p53 suppression and non-transforming *MYCL*, in addition to the usual reprogramming genes, *POU5F1, SOX2, KLF4,* and *LIN28* were employed to reprogram the fibroblasts^27^. Three micrograms of combined episomal plasmids (Addgene 27077, 27078 and 27080) were electroporated into 6 × 10^5^ fibroblast cells by using a Nucleofector II device (Lonza) and an Amaxa NHDF Nucleofector kit (Lonza) according to the manufacturer's instructions^28^. The electroporated cells were allowed to recover for 2 to 4 days by culturing in the above conditions. Cells (2 × 10^5^) were placed into 100 mm dishes previously coated with Matrigel (BD Bioscience). The following day the culture medium was switched to mTeSR1^29^ (STEMCELL Technologies). Colonies emerged about 14 days post-transduction. Colonies were mechanically isolated around day 20 and expanded under feeder free conditions in mTeSR1 medium on a Matrigel coated substratum.

### Neural progenitor cell (NPC) differentiation

NPC lines were generated from iPSCs through monolayer culture protocol using STEMdiff Neural Induction Medium (StemCell Technologies), following the manufacturer’s instructions. Briefly, 2 × 10^6^ iPSCs were transferred onto Matrigel-coated d35 plate (day 0) with Neural Induction Medium. The medium was refreshed daily, and the cells were passaged when they were confluent (day 7–9). By that time, the cells showed robust PAX6 expression and extensive reductions of pluripotent markers (POU5F1, NANOG). After passage (p), PAX6 expression in the entire cell population was further improved and the cell doubling time was determined by cell counting between p1 and 2 (day 8–15). RNA preparations and cells for immunostaining were collected between p1 and p4 (day 12–22) to ensure that both control and MAND iPSC lines were equally and fully differentiated into NPC stage and avoided any undifferentiated cell to be included (Supplementary Table 1).

### Immunostaining

Cells were grown on Lab-Tek II 4-well chambered cover glass (Nunc) coated with Matrigel. After fixing the cells in 4% paraformaldehyde in PBS for 10 min and permeabilizing them in 1.0% Triton X-100/PBS for 30 min, coverslips were placed in 5% (v/v) goat serum/5% w/v BSA in PBS for 1 h. Cells were then incubated with appropriately diluted primary antibodies (Supplementary Table 2) overnight at 4 °C. Secondary antibody staining was performed with either Alexa Fluor 568-, 647- or 488-labeled detection reagents (goat anti-rabbit, goat anti-mouse antibodies; Molecular Probes) at 1:300 dilution (Supplementary Table 2). Images were captured under an Olympus IX70 inverted microscope with an ORCA-AG CCD camera (http://www.biotech.missouri.edu/mcc/Olympus.html).

### Quantitative RT-PCR

This analysis was performed similar to previously reported^6,30^. Briefly, RNA was isolated from control and patient NPC lines using RNeasy Plus Mini Kits (QIAGEN) according to manufacturer’s instructions. All samples were treated with DNAse I. RNA was quantified using the NanoDrop ND-100 Spectrophotometer. First-strand cDNA synthesis was carried out with qScript cDNA SuperMix (Quanta Biosciences) according to manufacturer’s instructions. For quantitative real-time PCR, predesigned Taqman MGB (minor groove binder) probes from Assays-on-Demand Gene Expression Products (Life Technologies) were used for all genes (see Supplementary Table 3). All samples were run in triplicate in 10 μL reaction volumes. PCR conditions were the default setting of the ABI Prism 7900 HT Sequence Detection System (Life Technologies). The cycle threshold (Ct) was determined during the geometric phase of the PCR amplification plots, as recommended by the manufacturer. Relative differences in transcript levels were quantified with the ΔΔCt method with *GAPDH* as an endogenous control and were compared between control and MAND cell lines. Statistical analysis for gene expression differences by one-way ANOVA was performed with Prism 8.4.2 (GraphPad Software, graphpad.com).

### RNA-seq transcriptome analysis

RNA was isolated from NPC using RNeasy Plus Mini Kits (QIAGEN). The quantitation and quality control of RNA from all samples were performed on a Fragment Analyzer (Advanced Analytical), and cDNA libraries were constructed by standard methods (Illumina TruSeq mRNA stranded kit) with index adapters (Illumina TruSeq indexes). All libraries were sequenced as paired-end, 50 base read length on an Illumina HiSeq 2500 with an average read count of 21–28 million reads per sample at University of Missouri DNA core (dnacore.missouri.edu/). Once reads were obtained, all reads were analyzed for quality using FastQC (http://www.bioinformatics.babraham.ac.uk/projects/fastqc/), aligned to human genome GRCh38 using STAR^31^ and counted using FeatureCounts^32^. The resulting gene-wise count matrices were used for downstream analyses. Differential gene expression analysis was conducted using DeSeq2^33^ comparing MAND NPC lines with control NPC lines. Of the 59,074 transcripts analyzed, 19,801 passed all DeSeq2 quality filters and were given a *p* value. Genes were considered differentially expressed if they reached a corrected *p* < 0.005 using a Benjamini–Hochberg corrected false discovery rate (FDR) α < 0.005. Data for these experiments are freely available at the Gene Expression Omnibus (GSE141835). The significantly differentially expressed gene set (498 genes shown in Supplementary Data 1) was cleaned and converted to HGNC approved gene names. The list was further edited for unmatched terms and synonyms resulting in a list of 468 genes (Supplementary Data 3, Tab 1).

### Gene expression network analysis: biological processes and enrichment

To identify the prevalent biological pathways affected in MAND due to a 2q23.1 deletion, we used four independent pathway analysis databases (BIOCARTA, KEGG^34^, PANTHER^35^ and REACTOM^36^) to incorporate and assess the 468 differentially expressed genes (significance cut off *p* = 0.05). Since each of these approaches utilize different methodologies and algorithms, we conducted each analysis separately to provide the most comprehensive approach to pathway interrogation. We also utilized Gene Set Enrichment Analysis (GSEA)^37^ with default parameters for Gene Ontology (GO) analysis of biological processes, molecular functions and pathways. To identify the enrichment of the 468 differentially expressed genes by chromosomal location, we utilized Enrichr^38^. This enrichment analysis relied on ranking the list of genes based on statistical significance with the default settings of GSEA^39^.

### Comparison of MAND and ASD biological processes and pathway analyses

We first compared the MAND differentially expressed genes list (Supplementary Data 3, Tab 1) to the SFARI database gene list (Supplementary Data 3, Tab 2), which is a well-curated list of 913 genes, then we expanded this list to be inclusive for HGNC synonyms to a final list of 944 genes. We compared the two lists to identify the overlapping genes (Supplementary Data 3, Tab 3) and then sought to compare the biological processes which were represented by each gene list using the ToppFUN tool^40^ (Supplementary Data 3, Tab 4). We limited our analysis to GO: Biological Process with a Bonferroni correction for multiple testing, a statistical *p* value cutoff of 0.05, and limited genes to 1 < n < 1500. We captured the top 50 enriched biological processes and compared them between the two lists (additional tables in the Tab 4). Finally, we investigated whether our MAND differentially expressed genes were involved in known ASD associated pathways (Supplementary Data 3, Tab 5). Pathways were visualized utilizing the Reactome FIViz application in Cytoscape^41^ (Fig. 4c).

## Results

### Identification of MAND (2q23.1 deletion syndrome) patients

Three individuals with 2q23.1 deletion syndrome were identified clinically by chromosomal microarray analysis (CMA) (Fig. 1a, Supplementary Table 1). Despite the differently sized deletions (Fig. 1a), all deletions include *MBD5* exon 1, containing the transcription start site, as well as the *MBD5* translation start site (located in exon 6), and as a consequence, all three patients have ~ 50% *MBD5* mRNA expression (*p* < 0.0001) resulting in haploinsufficiency (Fig. 1b). These three patients have phenotypes commonly observed in MAND, including developmental delay, intellectual disability, sleep disturbance, seizures, language delay, hypotonia, mild dysmorphic features, and ASD-associated behaviors (short attention span and stereotypic, repetitive behaviors), consistent with the most prevalent clinical features of 2q23.1 deletion syndrome^5^. Since the primary phenotypic concerns for patients with 2q23.1 deletion syndrome are neurodevelopmental, we took an approach to investigate the molecular mechanism(s) underlying these phenotypes by creating neural progenitor stem cells from fibroblast cell lines derived from skin biopsies from each patient (Fig. 1c).

### Generation and characterization of iPSC derived from MAND patient fibroblasts

**Figure 2:**
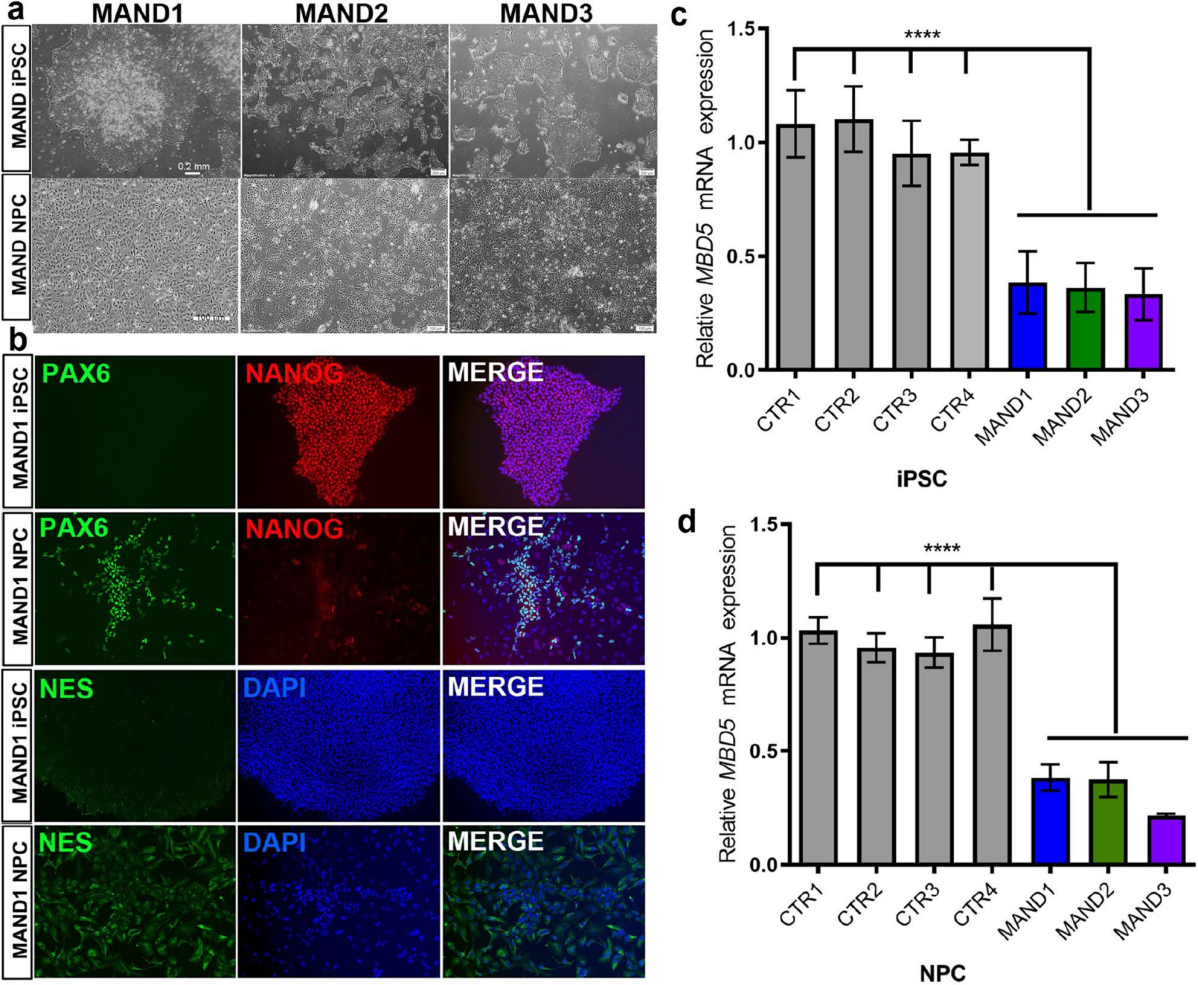
Generation of iPSC and NPC lines from individuals with MAND. (**a**) Bright-field images of undifferentiated iPSC lines from MAND1-3 (top row panel, l to r). Lower panel images are three NPC lines derived from MAND1-3 iPSC lines, respectively (l to r). After two weeks of neuronal induction, images were captured. Bars, 0.2 mm. (**b**) Immunostaining of MAND1 iPSC (1^st^ row) and MAND1 NPC (2^nd^ row) for neural progenitor cell marker PAX6 (1^st^ column) and pluripotent cell marker NANOG (2^nd^ column and 3^rd^ for merged). Immunostaining of MAND1 iPSC and MAND1 NPC for NES (3^rd^ and 4^th^ rows, respectively). (**c, d**) qRT-PCR analysis confirmed ~ 50% reduction of *MBD5* mRNA expression in MAND iPSC (**c**) and NPC lines (**d**) when compared with control iPSC and NPC lines, respectively. *p* < 0.0001. Quantification of the test gene (*MBD5*) was calculated relative to that of the housekeeping gene, *GAPDH*. All expression values were calculated relative to control levels set at 1.0. Error bars represent the SEM. ****p < 0.0001, n = 3 independent biological replicates performed in triplicate, GraphPad Prism 8.4.2 (graphpad.com).

Skin fibroblast cell lines from the three MAND patients and four normal controls were developed within one month from the time of the biopsy. The fibroblasts were expanded, stored, and then transduced to produce iPSCs in a feeder-free and defined culture system (Supplementary Fig. 1). The generated iPSC colonies expressed pluripotent markers, alkaline phosphatase, POU5F1 (also known as OCT4), NANOG, and TRA-1-60 (Supplementary Fig. 1). Programming efficiencies for MAND1-3 were 0.016, 0.4, and 0.022%, respectively. The successfully generated colonies were banked, and we utilized a subset of the iPSC lines to characterize MAND1-iPSC, MAND2-iPSC, MAND3-iPSC (Fig. 2a,b, Supplementary Fig. 2c), and CTR1-iPSC, CTR2-iPSC, CTR3-iPSC, and CTR4-iPSC (Supplementary Fig. 2a,b). Isolated DNAs from the iPSC lines were analyzed by chromosomal microarray (CMA) to confirm the molecular karyotype for each cell line was preserved and consistent with the original fibroblast line (Fig. 1a). Furthermore, we isolated RNA from these iPSCs and confirmed reduced *MBD5* mRNA expression in the MAND-iPSCs (Fig. 2c).

### Generation and characterization of NPCs derived from MAND-iPSCs

Using an established neural induction monolayer culture protocols (STEMCELL Technologies)^42,43^, MAND and control iPSC lines were differentiated into PAX6 expressing NPCs (Fig. 2a,b & Supplementary Fig. 2). Expression of the pluripotent stem cell marker NANOG and POU5F1, which was initially expressed in 100% of the iPSCs before the differentiation, had decreased to undetectable level. Another neural stem cell marker NES was expressed (Fig. 2b). Finally, DNA and RNA were isolated for CMA and RT-qPCR studies, respectively, to confirm cells maintained their original integrity during differentiation into neural lineage cells. CMAs of each NPC line were consistent with fibroblast and iPSC analyses, and RT-qPCR studies confirmed significantly reduced *MBD5* mRNA expression in the MAND-NPC lines (Fig. 2d).

### Global transcriptome analysis of MAND NPC reveals genome-wide alterations

**Figure 3:**
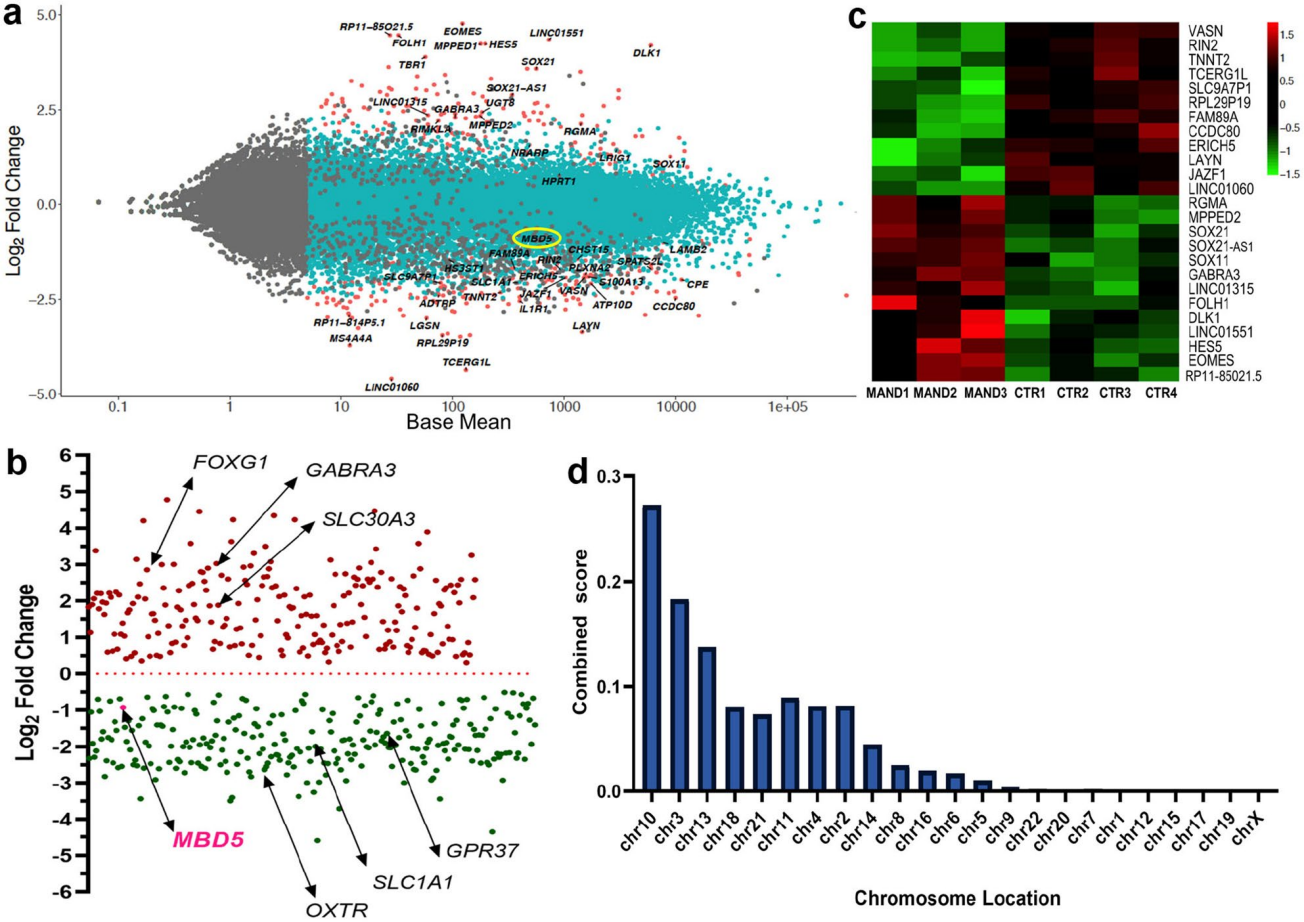
Global transcriptome analysis of MAND NPC reveals alterations of multiple genes. (**a**) Scatter plot of fold-change in gene expression in MAND NPC lines versus control NPC lines. The gene expression difference between MAND and CTR NPC lines are shown in log_2_ fold changes in y-axis. Genes more highly expressed in MAND NPC than in CTR NPC have a positive fold-change, while the down-regulated genes are illustrated with negative fold change. X-axis represents mean normalized expression. Significantly differentially expressed genes are shown in red. Some representative differentially expressed genes are indicated, including *MBD5* (circled in yellow). (**b**) MAND-associated 2q23.1 deletion results in both positive and negative effects on gene expression. The 468 differentially expressed genes are plotted based on their log_2_ fold changes in y-axis. Arrows point to *MBD5* and other genes that are associated with autism spectrum disorder (ASD). (**c**) Heatmap for top 25 differentially expressed genes; the 12 most downregulated and 13 most upregulated genes in the MAND NPC compared with control NPC lines are shown. (**d**) The 468 differentially expressed genes are plotted based on chromosome region. Based on the combined score (Supplementary Data 2, Tab 1), the greatest number of differentially expressed genes were on chromosomes 10, 3, 13, 18, and 21.

In order to investigate the genes, pathways, and biological processes that are disrupted due to *MBD5* haploinsufficiency that might provide insight into the etiology of this complex neurodevelopmental condition, we performed RNA-seq analysis on these established NPC lines. RNA-seq analysis (n = 3 MAND-NPCs, n = 4 CTR-NPCs) initially identified 498 differentially expressed genes in MAND-NPCs (Fig. 3a). From these 498 genes, there was an even distribution of upregulated and downregulated genes, confirming that MBD5 can act as a positive and negative regulator of gene expression (Fig. 3b). The top differentially expressed (up and down) genes in MAND-NPCs compared to the Control-NPCs are depicted in a heat map (Fig. 3c). The 228 genes up-regulated in MAND-NPCs (Fig. 3b, see Supplementary Data 2 for complete list) include those coding for a variety of transcription factors, including FOXG1* and other F-Box, T-Box, SOX, and homeobox genes; transmembrane proteins (transporters, receptors), including GABRA3* and SLC30A3*; ligands (*WNT7B*, *FGF9*); and non-coding RNAs (*VASN, RIN2*, *LINC01551, SOX21-AS1).* The 270 down-regulated genes (Fig. 3b, see Supplementary Data 1 for complete list) contained transcription regulators, including MBD5*, *FOXQ1*, *MSX2, GATA2*; transmembrane proteins: SLC1A1*, GPR37*, OXTR*, *FZD10*; and ligands: *NTF4, TGFB2, SMAD7*. Gene names with asterisk* are in SFARI Gene (https://www.sfari.org/resource/sfari-gene/) [version of June 20, 2019 released]. The initial 498 genes were assessed and converted to unique HGNC approved gene names, resulting in 468 unique genes, which were then used for all downstream analyses. Genome-wide expression differences as a consequence of the 2q23.1 deletion were examined using Enrichr, which sorted the 468 differentially expressed genes by their chromosome region and location. The chromosomes with the greatest number of differentially expressed genes were chromosomes 10, 3, 13, 18, 21, respectively (Fig. 3d, see Supplementary Data 2, Tab 1 for complete list of genes in the chromosome locations).

### MAND is associated with alterations in multiple biological processes and pathways

**Figure 4:**
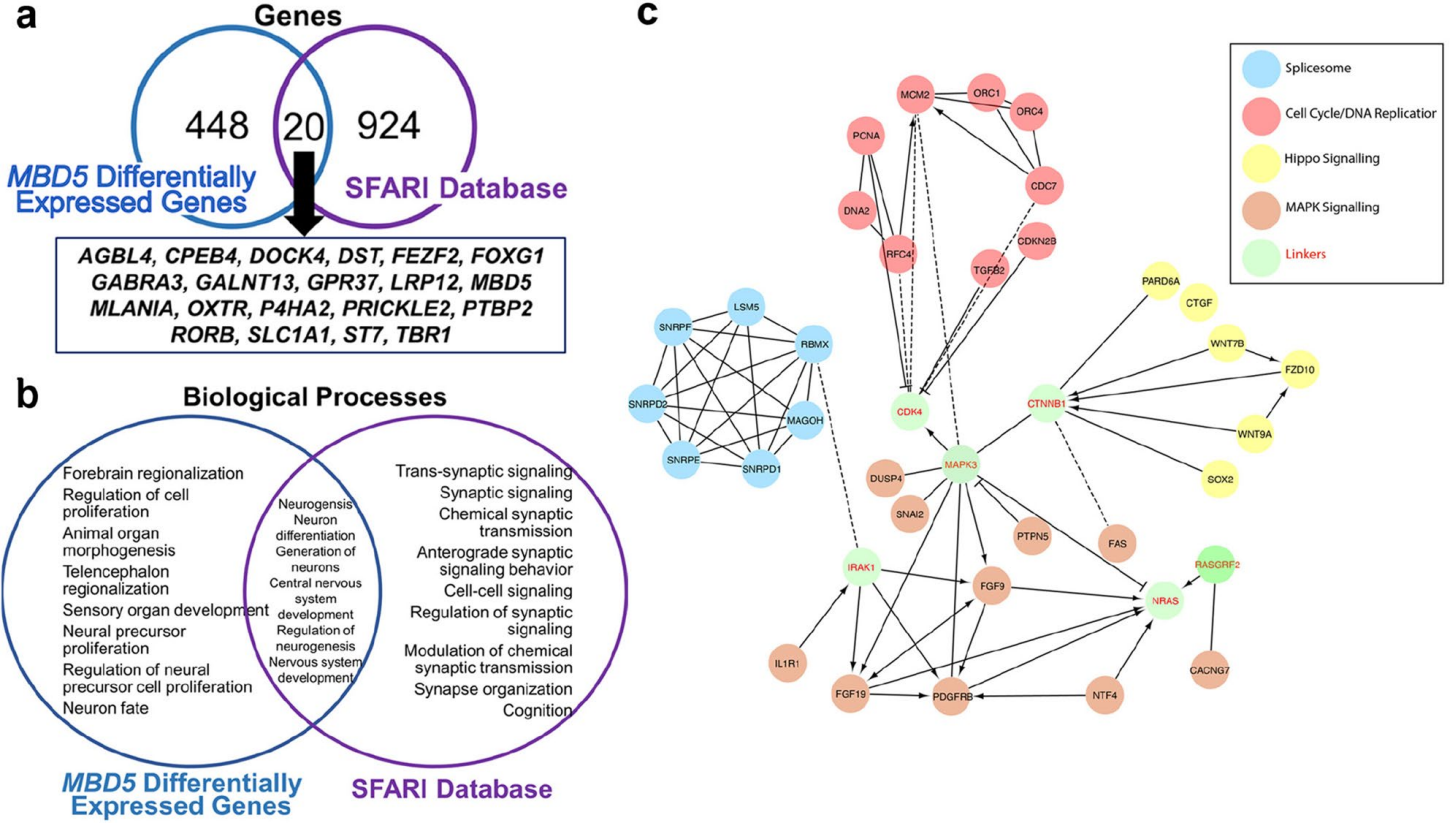
Altered cellular pathways and biological processes overlap between MAND and ASD. (**a**) Differentially expressed genes identified in MAND NPC RNA-seq data were compared to the syndromic and non-syndromic ASD genes in the SFARI database. The genes which are differentially expressed in MAND NPCs show overlap with 20 ASD associated genes and biological processes. In addition, the two gene lists also have their own unique biological processes. (**b**) Biological processes affected in MAND NPCs overlap those also enriched the SFARI dataset. Utilizing ToppGene, we see significant enrichment for multiple cellular and developmental processes, with overlap with central nervous system development, neurogenesis, neuron differentiation, generation of neurons, and nervous system development with the SFARI gene set. (**c**) Enrichment of ASD-associated pathways in MAND NPCs. Utilizing DAVID, significantly differentially expressed genes in MAND NPC are enriched in the following KEGG Pathways: spliceosome, MAPK signaling, cell division/DNA replication and WNT signaling. The solid and broken lines represent direct and indirect interactions, respectively. The arrow indicates activation, while a T head shows repression in the interaction.

To identify the biological processes that are affected by 2q23.1 deletions in MAND NPC, we utilized Gene Set Enrichment Analysis (GSEA), revealing high enrichment in these 5 categories: organ morphogenesis (*p* = 1.24e^−18^), regulation of cell population and proliferation (*p* = 3.07e^−16^), neurogenesis (*p* = 3.32e^−16^), neuron differentiation (*p* = 7.61e^−15^) and central nervous system development (*p* = 1.52e^−14^) (Supplementary Data 2, Tab 4). Performing GO analysis with the differentially expressed genes results showed that most genes dysregulated as a consequence of 2q23.1 deletion were involved in transcription regulatory region DNA binding (GO: 1990837 and 0044212) (Supplementary Data 2, Tab 3). To identify the key pathways impacted by the 468 differentially expressed genes, we ran our differential gene list through BIOCARTA and PANTHER pathway databases. Overall, our gene set was enriched in pathways involved in signal transduction, neuronal system, developmental biology, hemostasis, immune system, and disease (Supplementary Fig. 4). Key pathways that contained MAND differentially expressed genes were TGFβ and Hippo signaling pathways, receptor signaling by FGF and tyrosine kinases, DNA replication, and cell cycle (Fig. 4c, Supplementary Data 3, Tab 5).

### Transcriptional impact of MAND-associated 2q23.1 deletion on expression of autism-associated genes

We queried if there was overlap between the differentially expressed genes identified via RNA-seq in MAND NPCs and those genes known to be associated with autism. We compared MAND RNA-seq data to syndromic and non-syndromic ASD genes from SFARI (https://www.sfari.org/resource/sfari-gene/) (Fig. 4a, Supplementary Data 3, Tab 1–3). Genes differentially expressed in MAND NPCs show overlap with 4.3% of the ASD associated genes from SFARI (n = 20). We then compared our list with the SFARI autism gene set utilizing ToppCluster^40^. This analysis revealed striking significant overlap between the two gene lists on biological processes, which are in keeping with the neurological phenotype and ASD, and include forebrain and telencephalon regionalization, neuron fate commitment, and forebrain development (Fig. 4b). Interestingly, processes unique to each gene list were also identified, which may be an area for future studies of pathogenesis for each genetic subgroup. Finally, we discovered that several MAND differentially expressed genes are also involved in overrepresented pathways commonly seen in ASD^18,44,45^, including Hippo (8 genes identified in the pathway, i.e. n = 8), MAPK (n = 11), DNA replication/cell cycle (n = 11), and spliceosome formation pathways (n = 7) (Fig. 4c, Supplementary Data 3, Tab 5), providing additional biological connections between MAND and ASD.

### *FOXG1* expression is significantly increased in MAND NPC

**Figure 5:**
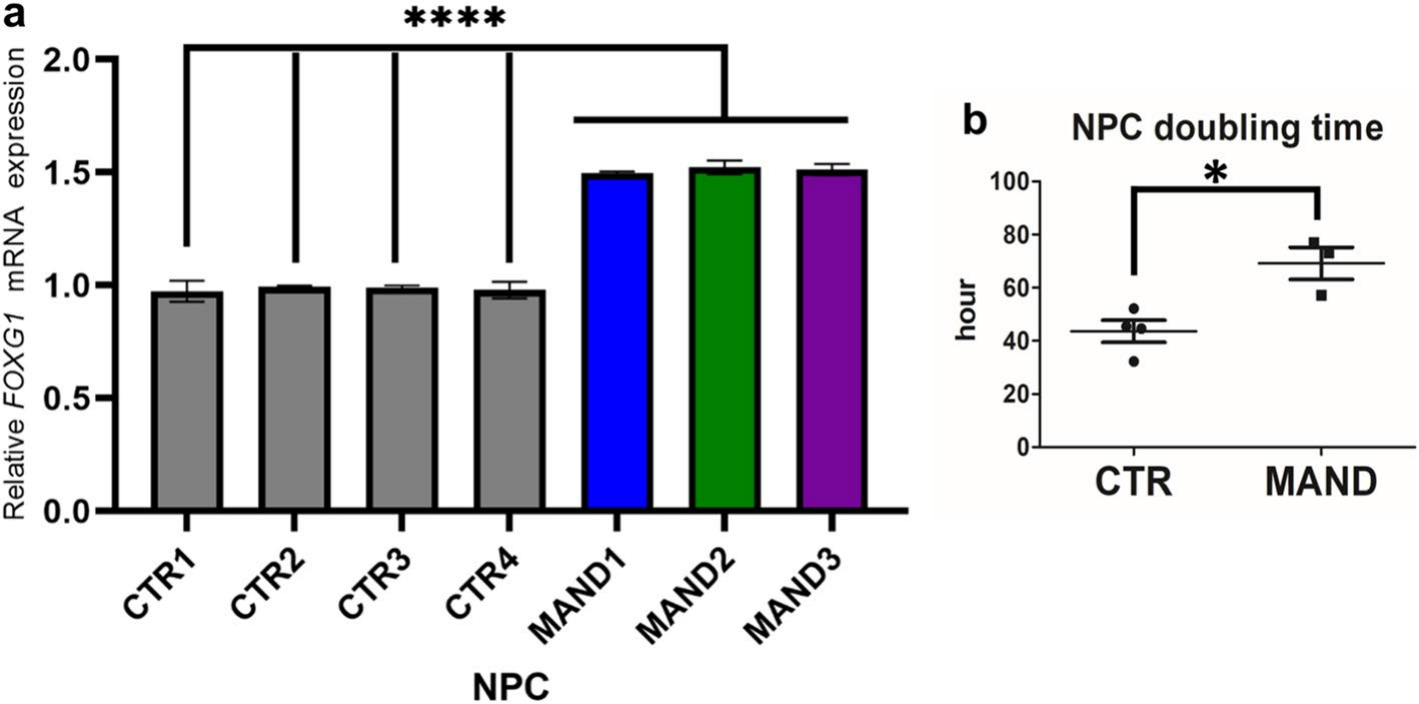
MAND-associated 2q23.1 deletion including *MBD5* results in de-repression of *FOXG1.* (**a**) Altered expression of *FOXG1* in MAND NPCs. Quantitative real-time PCR was performed on MAND NPC and control NPC lines. Levels of *FOXG1* mRNA were calculated relative to that of the housekeeping gene *GAPDH*. All expression values were calculated relative to control levels set at 1.0. Error bars represent the SEM. ****p < 0.0001, n = 3 independent biological replicates performed in triplicate. (**b**) Comparison of cell doubling time between MAND NPC and control NPC lines. Error bars represent the SEM. *p = 0.0152, GraphPad Prism 8.4.2 (graphpad.com).

In this study, MAND NPC RNA-seq data revealed significantly increased *FOXG1* mRNA levels over the controls, which was confirmed by RT-qPCR (Fig. 5a). *FOXG1* is essential for the normal development of the telencephalon, which is one biological process that is highly enriched in the MAND NPC data set (Supplementary Fig. 3 and Fig. 4b)^46,47^. Pathogenic variants in *FOXG1* are associated with autosomal dominant Rett syndrome, congenital variant [MIM 613454], which is characterized by normal birth but development of progressive microcephaly, severe hypotonia, midline stereotypic activities typical of classic Rett syndrome, no spoken language, and electroencephalography (EEG) abnormalities. *MBD5* (2q23.1 deletion) and *FOXG1* have each been coupled with Angelman syndrome-like (AS-L) cohorts and Rett syndrome-like cohorts (RS-L)^48^. Further, duplication of *FOXG1* is associated with West syndrome, a neurodevelopmental syndrome that includes epilepsy, developmental delays, and severe speech impairment^46,49,50^, similar to the phenotype observed in MAND. Increased *FOXG1* expression was also noted in ASD proband forebrain organoids over the control organoids, and down regulation of *FOXG1* in the organoid rescued the ASD-associated phenotype^51^. We compared the growth of MAND NPCs to CTR NPC and found that the doubling time of MAND NPC was significantly slower than CTR NPC (Fig. 5b). Overall, 2q23.1 deletion results in de-repression of *FOXG1* and other genes, including *SOX11*, *EZH2, EED, ETV5,* and *KDM1A,* which all function as chromatin modifiers and are implicated in neurogenesis (Supplementary Data 2, Tab 4), further connecting this network of epigenetic modifiers and their critical roles in neurodevelopment.

## Discussion

Investigation and understanding of neurodevelopmental disorders has been fueled by the development of iPSC from patients with these complex genetic conditions^14^. In this study, we generated and characterized iPSCs and NPCs that were derived from MAND (2q23.1 deletion syndrome) patients with overlapping phenotypes of ID and autism. By utilizing global transcriptome analysis, we were able identify differentially expressed genes in early neuronal development that are associated with pathways and genes common to ASD. These findings support previous knowledge about the functional role of MBD5 but also provide new insights into the consequences of chromosome 2q23.1 deletion in the regulation of numerous pathways that when altered contribute to the phenotype in 2q23.1 deletion syndrome.

In this study, cells were directly collected from patients with MAND and converted to iPSCs and NPCs. This approach allowed us to recapitulate the gene dosage effects in early neuronal developmental stages in human cells. There are advantages of our model compared to previous models to understand MAND. A previous study using an *Mbd5* gene-trap mouse model showed abnormal behavior and cognitive impairment^9^; however, this *Mbd5* gene-trap model is not representative of human 2q23.1 deletion syndrome. Another study utilized primary human neural precursor cells that were treated by a short hairpin RNA (shRNA) to suppress *MBD5* expression^52^; however, this methodology resulted only in a single cell line with reduced *MBD5* expression, limiting the analysis of data. In the most recent study, Seabra et al. (2020) generated CRISPR-derived neurons with mutation of *MBD5* and then conducted RNA-seq^53^. However, *MBD5* was not sufficiently dysregulated in the study; thus, the data presented are not truly representative of MAND^53^. The present study utilizing patient-derived NPC provides a representative disease model, consistent with other recent ASD studies that have shown the advantages of using iPSC technology for the study of genetic impact on human early neuronal developmental stage^54^.

Previous studies have shown that 2q23.1 deletions are critical for neurodevelopment and that *MBD5* haploinsufficiency is responsible for phenotypes, such as seizures, sleep, speech impairment, and autism^5–7,30,55^. These studies fell short in identifying the exact targets of MBD5 and the perturbed biological processes and pathways consequent to *MBD5* haploinsufficiency that could explain these neurodevelopmental phenotypes. In our study, we recognize that the dysregulated genes and pathways identified may not necessarily be due to direct effects of *MBD5* haploinsufficiency alone and could be due to indirect, downstream effects of reduced *MBD5* expression or may be due to reduced expression of other deleted gene(s) in these patient samples. Additional molecular studies are required to understand the effect of *MBD5* haploinsufficiency specifically on these dysregulated genes. Differentially expressed genes in NPC of MAND patients included genes associated with other neurodevelopmental disorders (Figs. 3, 4). A study of pathogenic de novo mutations that link to intellectual disability identified mutations in genes identified in our data set: *FOXG1, MBD5, TBR1*, *GABRB3* and others^56^ (Supplementary Data 3, Tab 3). By the Gene Ontology (GO) enrichment analysis of the transcriptome data, biological functions of neuron development, brain development and cellular processes were altered in the cohort in alignment with the neurodevelopmental phenotype present in these individuals (Supplementary Fig. 3).

In this study, we took advantage of the ability to derive NPCs from MAND patient cells and RNA-seq analysis to investigate the role of MAND on ASD-associated genes and pathways (Figs. 3, 4). Studies of ASD patient-derived neuronal cells have demonstrated altered gene expression and dysregulation of pathways such as Hippo, MAPK, DNA replication/cell cycle, and spliceosome formation pathways^57,58^. While our study may have been hindered by small sample size, our analysis using the SFARI database identified 20 genes (4.3%) linked to ASD with altered gene expression due to 2q23.1 deletion, highlighting the commonalities between the MAND and ASD (Fig. 4a). This percentage is similar to the enrichment for SFARI genes in other key ASD RNA-seq studies^44,57–59^. These genes could be the key to understanding the connection between MAND and autism. Further, six key biological processes are shared between our data set and genes with known association to ASD (Fig. 4b), suggesting that these biological processes are key to understanding the overlapping phenotypes between MAND and ASD. Further study of these biological processes is needed to more clearly elucidate the physiological mechanisms that are altered.

**Figure 6:**
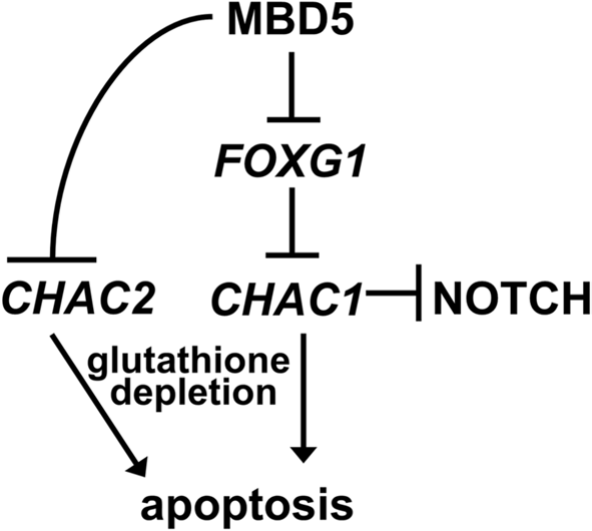
Schematic model of the possible role of MBD5 in regulation of glutathione, apoptosis, and Notch signaling pathway.

The MAND-derived NPC RNA-seq data revealed differentially expressed genes previously linked to neurodevelopmental disorders. Some of the most interesting connections in phenotypically similar neurodevelopmental disorders include the *FOXG1* dosage-linked associations with Rett syndrome and West syndrome. Our findings in this study indicate that MBD5 either directly or indirectly regulates *FOXG1* expression in an early neurogenesis stage (Fig. 5). Mutation and/or altered expression of *FOXG1* have been shown to cause an autosomal dominant form of Rett syndrome^60^, associated with ASD^59^, West syndrome^50^, and schizophrenia^61^. Since *FOXG1* is a dosage sensitive gene, its overexpression would likely affect proper brain development and cognitive functions. Furthermore, this may represent a possible route for convergent phenotypes between MAND and other syndromes, including West syndrome associated with duplication of *FOXG1*^50^. The slow growth of MAND NPCs (Fig. 5b) may represent a link between *FOXG1* dysregulation and improper glutathione synthesis. *CHAC2* expression is also altered in our dataset (Supplementary Data 1). CHAC2 is thought to be essential for glutathione maintenance^62,63^. Interestingly, FOXG1 plays a role in the transcriptional repression of *CHAC1* which is in the same family as *CHAC2* and also plays a role in glutathione maintenance (Fig. 6)*. CHAC1* is thought be a pro-apoptotic factor and inhibitor of NOTCH signaling in glioblastoma cell lines^64^. Further, FOXG1 is thought to play a role in glutathione maintenance and glutathione is thought to regulate cell proliferation^65,66^. Future molecular studies to assess MBD5 regulation of *FOXG1* is necessary especially in relation to glutathione synthesis and Notch signaling (Fig. 6).

Here, our study of MAND with human neural in vitro cultures through patient-derived NPC demonstrates *FOXG1* upregulation (Fig. 5), as well as perturbed expression of genes implicated in brain development, especially forebrain regionalization (Fig. 4b), and affected genes significantly overlap with ASD associated genes (Fig. 4a). These findings expand our knowledge about gene network differences and possible interactions between these related disease pathways (Figs. 4c, 6). Such findings may lead to potential connections between overlapping neurocognitive and neuropsychiatric phenotypes and associated risk factors, such as other chromatin modifiers and epigenetic factors that link to ASD. We expect these in vitro models will nurture hypotheses toward therapeutic interventions^67,68^.

## Web resources

The URLS for data present herein are as follows:

AutismKB, http://autismkb.cbi.pku.edu.cn/.

Cytoscape, http://www.cytoscape.org/.

DAVID Bioinformatics Resources 6.7, http://david.abcc.ncifcrf.gov/.

Enrichr, http://amp.pharm.mssm.edu/Enrichr/.

GSEA (Gene Set Enrichment Analysis), https://www.gsea-msigdb.org/gsea/index.jsp.

KEGG, http://www.genome.jp/kegg/.

Online Mendelian Inheritance in Man (OMIM), http://www.omim.org/.

PANTHER, http://www.pantherdb.org.

SFARI Human Gene Module (accessed February 18, 2020), https://gene.sfari.org/autdb/HG_home.do.

ToppCluster https://toppgene.cchmc.org/.

UCS Genome Browser, http://genome.ucsc.edu.

## Supplementary Information

Supplementary Information 1.

Supplementary Information 2.

Supplementary Information 3.

Supplementary Information 4.

## Data Availability

The RNA-seq data are available in the Gene Expression Omnibus (GSE141835).
