# Supplementary material for: Transcriptome analysis of MBD5-associated neurodevelopmental disorder (MAND) neural progenitor cells reveals dysregulation of autism-associated genes
Source: Sci Rep. 2021 May 28;11:11295. doi: 10.1038/s41598-021-90798-z (PMC8163803; doi:10.1038/s41598-021-90798-z)
Supplement: Supplementary file 1 — Supplementary Information 1. [file 41598_2021_90798_MOESM1_ESM.docx]

**Supplementary Information**

**Transcriptome analysis of *MBD5*-associated neurodevelopmental disorder (MAND) neural progenitor cells reveals dysregulation of** **autism-associated genes.**

**Authors**

Sureni V. Mullegama^1^, Steven D. Klein^2^_,_ Stephen R. Williams^3^_,_ Jeffrey W. Innis^4^, Frank J. Probst^1^, Chad Haldeman-Englert^5^**,** Julian A. Martinez-Agosto^2^, Ying Yang^6^, Yuchen Tian^7^, Sarah H. Elsea^1*^ and Toshihiko Ezashi^7*^

**Author affiliations**

^1^Department of Molecular and Human Genetics, Baylor College of Medicine, One Baylor Plaza, Houston, TX, 77030, USA. ^2^Department of Human Genetics, David Geffen School of Medicine, University of California, Los Angeles, Los Angeles, California, USA. ^3^10X Genomics, San Francisco, CA, USA. ^4^Departments of Human Genetics, Pediatrics and Internal Medicine, University of Michigan, Ann Arbor, MI 48109, USA. ^5^Mission Fullerton Genetics Center, Asheville, NC, 28803, USA. ^6^Department of Molecular Pharmacology and Physiology, University of South Florida, Tampa, Florida 33612, USA. ^7^Division of Animal Sciences and Bond Life Sciences Center, University of Missouri, Columbia, Missouri 65211, USA.

**Current affiliations:**

Sureni V Mullegama, GeneDx, Gaithersburg, MD, USA

Steven D Klein, Department of Pediatrics, Children’s Hospital of Philadelphia, Philadelphia, PA 19104, USA & Department of Medical Genetics, Children’s Hospital of Philadelphia, Philadelphia, PA 19104, USA

***Correspondence should be addressed to:**

Toshihiko Ezashi, DVM, PhD, Email: ezashit@missouri.edu

Sarah H. Elsea, PhD, FACMG, Email: elsea@bcm.edu

**Supplementary Table 1.** Sample demographics for control and MAND cell lines

| **Sample^a^** | **ID** | **age** | **sex** | **reference** | **CMA** | **doubling time assay** | **Cell and RNA collections** |
| --- | --- | --- | --- | --- | --- | --- | --- |
| **CTR 1** | SB001 | 42 y | F |  | normal with polymorphic variants | p1, day 8 | p2, day 14 |
| **CTR 2** | CTL5 (#5-1) | 39.5 wk | F | **1** | normal with polymorphic variants | p1, day 9 | p2, day 12 |
| **CTR 3** | GM#05659 | 1 y | M |  | normal with polymorphic variants | p1, day 10 | p4, day 17 |
| **CTR 4** | SMS451 | 17 y | M |  | normal with polymorphic variants | p1, day 12 | p3, day 18 |
| **MAND 1** | GM#24585 (SMS388) | 3 y | F | **2,3** | 46,XX.arr[hg19]2q22.3q23.1(148,345,483-149,080,197)x1,15q11.1q11.2(20,262,223-22,285,757)x1 | p2, day 13 | p2, day 22 |
| **MAND 2** | SMS447 | 10 y | M |  | 46,XY.arr[hg19]2q23.1(148,687,657-148,872,174)x1 | p1, day 15 | p2, day 17 |
| **MAND 3** | SMS456 | 8 yr | F | **2** | 46,XX.arr[hg19]2q22.3-2q23.3(148,326,568-151,757,065)x1 | p1, day 14 | p1, day 14 |

**^a^CTR** = Control

**References Supplementary Table 1.**

**1**. Sheridan MA, Yang Y, Jain A, Lyons AS, Yang P, Brahmasani SR, et al. Early onset preeclampsia in a model for human placental trophoblast. *Proceedings of the National Academy of Sciences*. 2019;116(10):4336-45. <https://www.ncbi.nlm.nih.gov/pmc/articles/PMC6410818/>

**2**. Mullegama SV, Elsea SH. Intragenic MBD5 familial deletion variant does not negatively impact MBD5 mRNA expression. *Molecular cytogenetics*. 2014;7(1):80. [PMC4243375](https://www.ncbi.nlm.nih.gov/pmc/articles/PMC4243375/).

**3**. Mullegama SV, Rosenfeld JA, Orellana C, van Bon BW, Halbach S, Repnikova EA, et al. Reciprocal deletion and duplication at 2q23.1 indicates a role for MBD5 in autism spectrum disorder. *Eur J Hum Genet*. 2014;22(1):57-63. [PMC3865402.](https://www.ncbi.nlm.nih.gov/pmc/articles/PMC3865402/)

**Supplementary Table 2.** Primary and secondary antibodies used in immunostaining.

| Antibody | Catalog # (Source) | Dilution | |
| --- | --- | --- | --- |
| POU5F1 | sc-5279 mouse (Santa Cruz) | | 1:100 |
| NANOG | ab109250 rabbit (Abcam) | | 1:100 |
| PAX6 | mouse monoclonal (DSHB) | | 1:50 |
| SOX1 | ab109290 rabbit (Abcam) | | 1:100 |
| NES (Nestin) | ab6320 mouse (Abcam) | | 1:100 |
| Alexa Fluor 488 Donkey Anti-Mouse IgG | A-21202 (Thermo/Life Technologies) | | 1:300 |
| Alexa Fluor 568 Donkey Anti-Rabbit IgG | A-10042 (Thermo/Life Technologies) | | 1:300 |
| Alexa Fluor 568 Donkey Anti-Mouse IgG | A-10037 (Thermo/Life Technologies) | | 1:300 |

**Supplementary Table 3.** TaqMan® Gene Expression Probe IDs for genes assessed in expression studies

| Gene | OMIM | Probe ID |  | |
| --- | --- | --- | --- | --- |
| *MBD5* | #156200 | Hs00289233_m1 | |  |
| *FOXG1* | #613454 | Hs01850784_s1 | |  |
| *GAPDH* | #138400 | Hs99999905_m1 | |  |


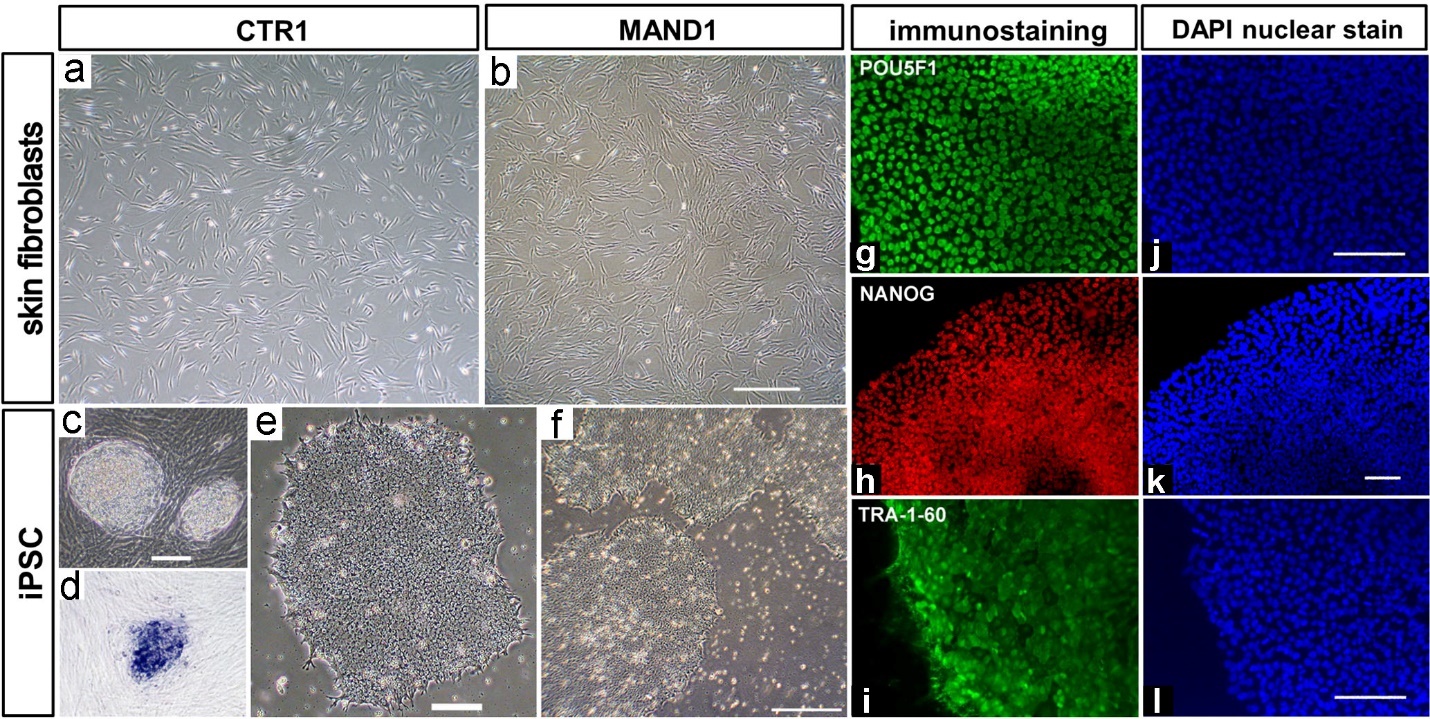


**Supplementary Figure 1. Generation of iPSC from skin fibroblasts of CTR1 and MAND1**. (**a, b**) Phase contrast micrographs of skin fibroblasts of CTR1 and MAND1, respectively. Scale bar, 500 µm. (**c**) Image of CTR1 iPSC colonies begin to emerge 17 days following plasmid transfection on the fibroblasts. Scale bar, 100 µm. (**d**) The CTR1 iPSC colonies express alkaline phosphatase visualized with blue color. (**e, f**) The iPSC colonies were isolated from the fibroblasts and expanded on feeder-free culture condition with serial passages. **e**; CTR1 iPSC with 200 µm bar, **f**; MAND1 iPSC with 500 µm bar. (**g**-**l**) The established CTR1 iPSC colonies were immunostained for POU5F1 (**g**, green), NANOG (**h**, red) and TRA-1-60 (**i**, green). (**j**-**l**) Nuclear stains with DAPI were captured at the same sites of **g**-**i**, respectively. Nuclear localizations of POU5F1 and NANOG transcription factors and cell surface localization of TRA-1-60. Bars are 200 µm in **g**-**l**.


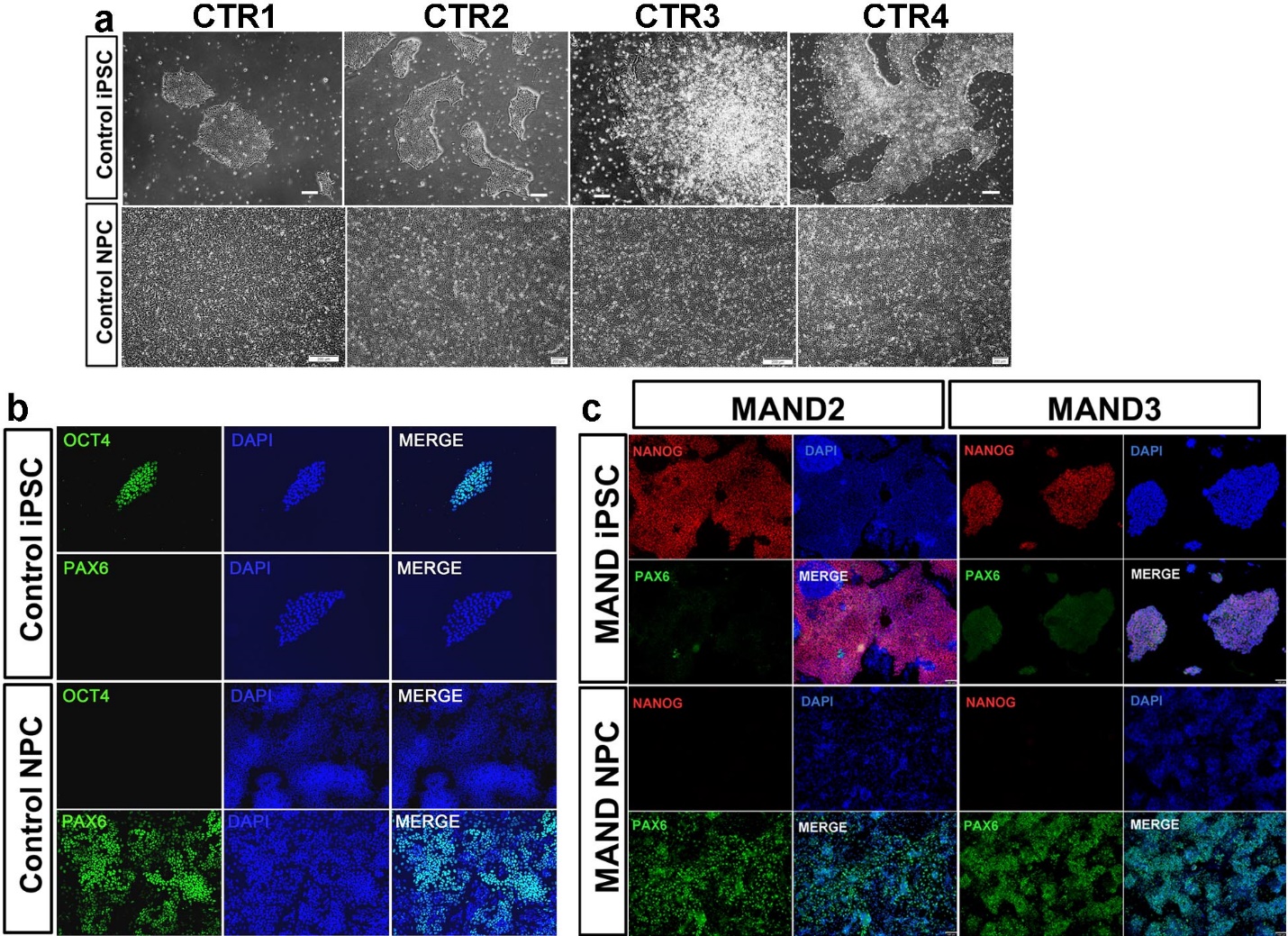


**Supplementary Figure 2. Differentiation of iPSC to neural progenitor cells (NPC)** (**a**) Bright-field images of undifferentiated iPSC (top) and NPC (lower) lines of four controls of CTR1, -2, -3 and -4 (l to r). Bars, 0.2 mm. Images of three NPC were captured after two weeks of neuronal induction. Bars, 0.2 mm. (**b**) Immunostaining of CTR1 iPSC and CTR1 NPC lines. The iPSC expressed pluripotent marker POU5F1 (OCT4, green, top panels) and were negative for neural progenitor marker PAX6 (2^nd^ row panels). Center panels show nuclear staining with DAPI, and right panels are merged images of immunostaining (green) and nuclear (blue) staining. The CTR1 NPC cells were immunostained with POU5F1 (OCT4, 3^rd^ panels) and PAX6 (4^th^ panels). (**c**) iPSC (top) and NPC (bottom) immunostainings of MAND2 (left) and MAND3 (right) lines. The iPSC lines expressed pluripotent marker NANOG (top panels) and were negative for neural progenitor marker PAX6 (2^nd^ panels). Nuclear staining with DAPI, and the merged images of immunostaining are also shown. The NPC lines were negative for NANOG (3^rd^ panels) and positive for PAX6 (4^th^ panels). Bars, 0.1 mm.

**
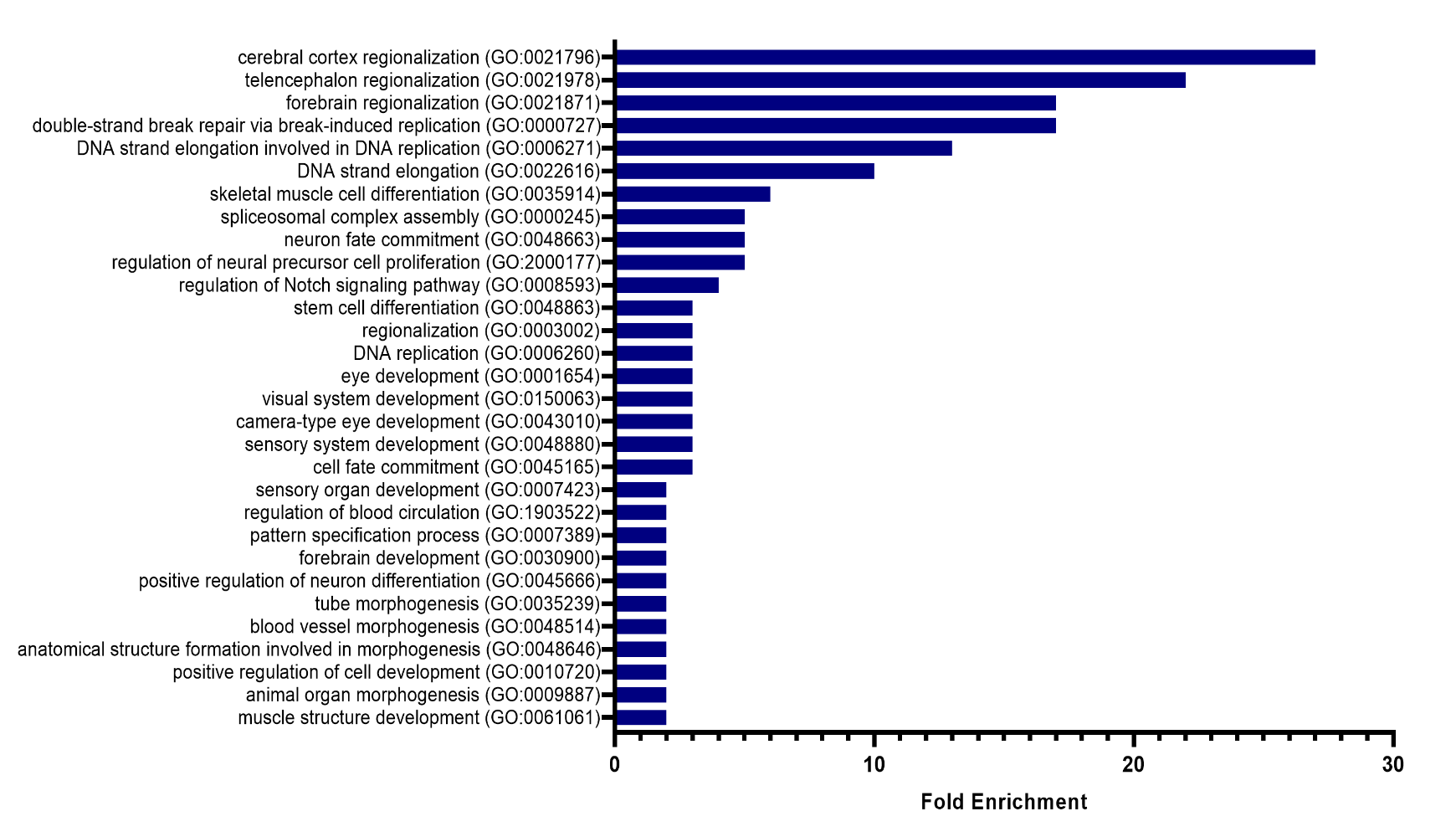
**

**Supplementary Figure 3.** **Multiple biological processes are affected in MAND NPCs.**  Top 30 of Gene Ontology biological processes altered in MAND NPC are shown. Bar plot showing the enrichment (−Log_10_(P-Value)) of biological processes by DAVID analysis. High enrichment for forebrain and telencephalon regionalization, central nervous system development, and head/brain development were identified in MAND NPC lines.


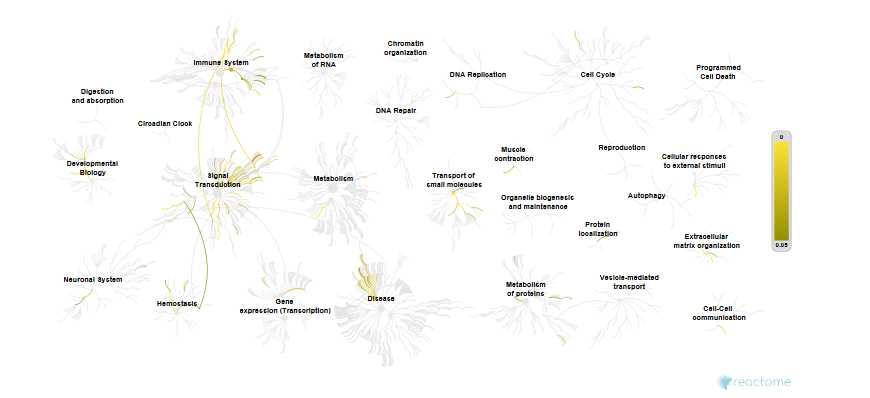
**Supplementary Figure 4. A Genome-Wide Overview of MAND Differentially Expressed Genes.** The center of each of the circular “bursts” is the root of one top-level pathway, for example, “DNA Repair”. Each step away from the center represents the next level lower in the pathway hierarchy. The color code denotes over-representation of the pathway in the 468 gene set. Light grey signifies pathways which are not significantly over-represented. The figure was generated with software in https://reactome.org/.


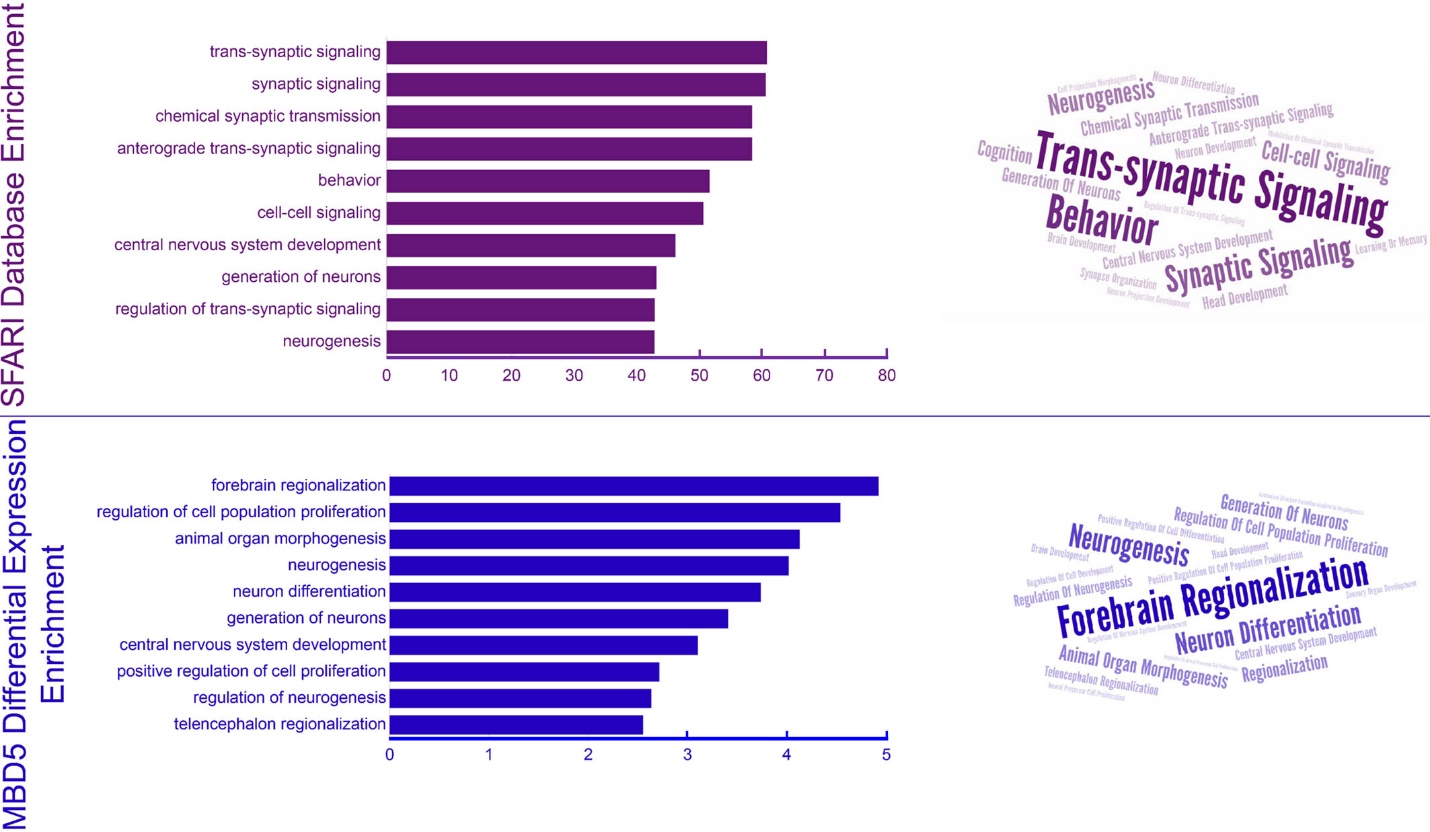


**Supplementary Figure 5.** **Biological processes affected in MAND NPCs compared to those enriched the SFARI dataset. Above.** Top 10 of enriched Gene Ontology biological processes that are overlapped with the SFARI autism gene database (purple). **Below.** The top 10 of enriched Gene Ontology biological processes specific to MAND NPC are shown (blue). Bar plot showing the enrichment (−Log_10_(FDR)) of biological processes by Topp FUN analysis.

**Legends for Supplementary Data excel files**

**Supplementary Data 1:** *MBD5* NPC RNA-Seq data of the top 498 dysregulated genes altered by *MBD5* deletions

**Supplementary Data 2:** Chromosome locations, biological, molecular functions and pathways altered by *MBD5* deletions in NPC RNA-Seq data

**Supplementary Data 3:** *MBD5* NPC RNA-Seq data set compared to SFARI gene data set and the biological functions and pathways that are shared between the data sets
